# Procyclidine Use in Long-Acting Injectable Antipsychotics

**DOI:** 10.1192/bjo.2025.10592

**Published:** 2025-06-20

**Authors:** Shalina Mitchell, Prabin Gautam

**Affiliations:** Kent and Medway NHS & Social Care Partnership Trust, Dartford, United Kingdom

## Abstract

**Aims:** Following a Serious Untoward Incident investigation into a patient death where blood tests revealed procyclidine overdose, significant concerns emerged regarding procyclidine prescribing practices. A notable discrepancy was identified between the Summary of Product Characteristics and British National Formulary regarding maximum daily procyclidine dosing, with evidence of 40 mg daily doses being administered. This raised questions about prescribing practices, particularly in patients receiving long-acting injectable antipsychotics. The incident highlighted the need to evaluate current prescribing patterns, assess protocol adherence, monitor effectiveness through GASS scores, and ensure appropriate documentation of procyclidine therapy.

**Methods:** A retrospective clinical audit was conducted within the Dartford, Gravesham, and Swanley Community Mental Health Team (DGS CMHT) from September 15, 2023, to January 7, 2024. The audit examined 36 patients receiving long-acting injectable antipsychotics. Data collection encompassed patient demographics, psychiatric diagnoses, antipsychotic medication details, and procyclidine usage patterns. The review also included an analysis of Glasgow Antipsychotic Side-effect Scale scores to assess medication side effects and an evaluation of procyclidine review documentation.

**Results:** Analysis showed that 39% (14/36) were prescribed regular procyclidine, with doses ranging from 5 mg to 30 mg daily. None of the patients were initiated on the recommended 2.5 mg TDS dosing regimen. Regular procyclidine reviews were documented for 92.9% of patients, and 64% demonstrated improvements in their GASS scores. The patient cohort was predominantly male (75%), with 44% aged between 55–64 years. The most common primary diagnoses were schizophrenia (69%) and bipolar disorder (25%), with other conditions accounting for 6% of cases.

**Conclusion:** The audit demonstrates both strengths and areas requiring improvement in procyclidine management at DGS CMHT. While the high rate of regular reviews (92.9%) and positive GASS score improvements (64%) indicate effective symptom management, non-adherence to recommended starting doses, inconsistent review intervals, and gaps in electronic documentation were important areas for improvement. Clear guidance, staff training, and updated clinical guidelines have been commenced to enhance patient safety, with a follow-up audit planned to monitor improvements.

